# Supplementary material for: What message appeal and messenger are most persuasive for COVID-19 vaccine uptake: Results from a 5-country survey in India, Indonesia, Kenya, Nigeria, and Ukraine
Source: PLoS One. 2022 Sep 21;17(9):e0274966. doi: 10.1371/journal.pone.0274966 (PMC9491563; doi:10.1371/journal.pone.0274966)
Supplement: S1 Table — (DOCX) [file pone.0274966.s001.docx]

**S1 Table. India relative risk ratios of ad preference by vaccine hesitancy status and participant characteristics using multivariable multinomial logistic regression modeling** (n=198)*

|  | ***Adjusted relative risk ratios (95% CI)*** | | | | | |
| --- | --- | --- | --- | --- | --- | --- |
|  | **Health Outcome**  **Peer** | **Economic**  **Healthcare provider** | **Economic**  **Peer** | **Social norm**  **Healthcare provider** | | **Social norm**  **Peer** |
| **Vaccine hesitancy** | | | | | | |
| Lower | Ref | Ref | Ref | Ref | Ref | |
| Higher | 1.42 (0.64, 3.15) | 1.60 (0.49, 5.16) | 1.49 (0.83, 2.67) | 0.98 (0.39, 2.46) | 3.44 (0.99, 11.94) | |
| **Age** | | | | | | |
| <40 | Ref | Ref | Ref | Ref | Ref | |
| 40+ | 0.47 (0.78, 2.82) | 1.88 (0.28, 12.78) | 1.46 (0.62, 3.35) | 1.80 (0.39, 8.38) | 1.59 | |
| **Gender** | | | | | | |
| Female | Ref | Ref | Ref | Ref | Ref | |
| Male | 0.93 (0.41, 2.11) | 0.44 (0.13, 1.48) | 0.65 (0.40, 1.07) | 0.76 (0.30, 1.96) | .51 (0.17, 1.55) | |
| **Education** | | | | | | |
| Secondary | Ref | Ref | Ref | Ref | Ref | |
| Bachelor’s Degree | 0.73 (0.21, 2.51) | 0.59 (0.09, 3.86) | 0.83 (0.46, 1.53) | 1.88 (0.33, 10.59) | 822567.8 | |
| Graduate Degree | 1.18 (0.33, 4.23) | 0.82 (0.12, 5.57) | 0.85 (0.39, 1.85) | 1.83 (0.30, 11.13) | 4.01 | |
| * Reference category: health outcome / healthcare provider ad | | | | | | |
