# Supplementary material for: What message appeal and messenger are most persuasive for COVID-19 vaccine uptake: Results from a 5-country survey in India, Indonesia, Kenya, Nigeria, and Ukraine
Source: PLoS One. 2022 Sep 21;17(9):e0274966. doi: 10.1371/journal.pone.0274966 (PMC9491563; doi:10.1371/journal.pone.0274966)
Supplement: S2 Table — (DOCX) [file pone.0274966.s002.docx]

**S2 Table. Indonesia relative risk ratios of ad preference by vaccine hesitancy status and participant characteristics using multivariable multinomial logistic regression modeling** (n=226)*

|  | ***Adjusted relative risk ratios (95% CI)*** | | | | | |
| --- | --- | --- | --- | --- | --- | --- |
|  | **Health Outcome**  **Peer** | **Economic**  **Healthcare provider** | **Economic**  **Peer** | **Social norm**  **Healthcare provider** | | **Social norm**  **Peer** |
| **Vaccine hesitancy** | | | | | | |
| Lower | Ref | Ref | Ref | Ref | Ref | |
| Higher | 1.90(0.88, 4.14) | 1.50 (0.46, 4.96) | 1.14 (0.49, 2.62) | **6.46 (1.78, 23.52)** | 0.85 (0.30, 2.38) | |
| **Age** | | | | | | |
| <40 | Ref | Ref | Ref | Ref | Ref | |
| 40+ | 0.18 (0.02, 1.54) | 3.00 | 0.27 (0.03, 2.41) | 0.27 (0.03, 2.53) | 2.90 | |
| **Gender** | | | | | | |
| Female | Ref | Ref | Ref | Ref | Ref | |
| Male | 0.90 (0.43, 1.90) | 1.61 (0.52, 4.94) | 1.05 (0.46, 2.41) | 1.23 (0.51, 3.00) | 1.08 (0.38, 3.05) | |
| **Education** | | | | | | |
| Secondary | Ref | Ref | Ref | Ref | Ref | |
| Bachelor’s Degree | 1.04 (0.49, 2.21) | 0.81 (0.23, 2.80) | 0.64 (0.27, 1.51) | 0.65 (0.25, 1.67) | 0.89 (0.32, 2.52) | |
| Graduate Degree | 0.28 (0.03, 2.74) | **5.16 (1.03, 25.79)** | 1.06 (0.21, 1.42) | 1.88 (0.42, 8.36) | 5.15 | |
| * Reference category: health outcome / healthcare provider ad | | | | | | |
