# Supplementary material for: What message appeal and messenger are most persuasive for COVID-19 vaccine uptake: Results from a 5-country survey in India, Indonesia, Kenya, Nigeria, and Ukraine
Source: PLoS One. 2022 Sep 21;17(9):e0274966. doi: 10.1371/journal.pone.0274966 (PMC9491563; doi:10.1371/journal.pone.0274966)
Supplement: S3 Table — (DOCX) [file pone.0274966.s003.docx]

**S3 Table. Kenya relative risk ratios of ad preference by vaccine hesitancy status and participant characteristics using multivariable multinomial logistic regression modeling** (n=170)*

|  | ***Adjusted relative risk ratios (95% CI)*** | | | | |
| --- | --- | --- | --- | --- | --- |
|  | **Health Outcome**  **Peer** | **Economic**  **Healthcare provider** | **Economic**  **Peer** | **Social norm**  **Healthcare provider** | **Social norm**  **Peer** |
| **Vaccine hesitancy** | | | | | |
| Lower | Ref | Ref | Ref | Ref | Ref |
| Higher | -0.18 (-1.17,0.80) | -0.66 (-2.06, 0.73) | 0.47 (-1.79, 2.74) | **-1.21 (-2.37, -0.06)** | 14.72 (-2174.50, 2203.96) |
| **Age** | | | | | |
| <40 | Ref | Ref | Ref | Ref | Ref |
| 40+ | 0.82 (0.22, 5.31) | 0.31 (-2.38, 2.44) | 0.74 (-1.72, 3.19) | 0.53 (-1.40, 2.44) | -14.60 (-3594.39, 3565.19) |
| **Gender** | | | | | |
| Female | Ref | Ref | Ref | Ref | Ref |
| Male | - 0.72 (-1.47, 0.36) | 0.11 (-1.12,1.34) | -0.57 (-2.07, 0.93) | -1.25 **(-2.30, -0.20)** | -0.40 (-1.85, 1.06) |
| **Education** | | | | | |
| Secondary | Ref | Ref | Ref | Ref | Ref |
| Bachelor’s Degree | 0.02 (-1.02, 1.05) | 0.16 (-1.60, 1.91) | -0.55 (-2.41, 0.93) | 0.26 (-1.13, 1.65) | -0.86 (-2.69, 0.97) |
| Graduate Degree | 0.76 (-0.74, 2.27) | 1.66 (-0.46, 3.77) | 0.96 (-2.61, 2.80) | 0.35 (-1.80, 2.50) | 0.63 (-1.70, 2.96) |
| * Reference category: health outcome / healthcare provider ad | | | | | |
