# Supplementary material for: What message appeal and messenger are most persuasive for COVID-19 vaccine uptake: Results from a 5-country survey in India, Indonesia, Kenya, Nigeria, and Ukraine
Source: PLoS One. 2022 Sep 21;17(9):e0274966. doi: 10.1371/journal.pone.0274966 (PMC9491563; doi:10.1371/journal.pone.0274966)
Supplement: S4 Table — (DOCX) [file pone.0274966.s004.docx]

**S4 Table. Nigeria relative risk ratios of ad preference by vaccine hesitancy status and participant characteristics using multivariable multinomial logistic regression modeling** (n=151)*

|  | ***Adjusted relative risk ratios (95% CI)*** | | | | |
| --- | --- | --- | --- | --- | --- |
|  | **Health Outcome**  **Peer** | **Economic**  **Healthcare provider** | **Economic**  **Peer** | **Social norm**  **Healthcare provider** | **Social norm**  **Peer** |
| **Vaccine hesitancy** | | | | | |
| Lower | Ref | Ref | Ref | Ref | Ref |
| Higher | 1.31 (0.39, 4.49) | 1.22 | 1.96 | 1.52 (0.15, 14.92) | 0.28 (0.05, 1.67) |
| **Age** | | | | | |
| <40 | Ref | Ref | Ref | Ref | Ref |
| 40+ | 0.96 (0.27, 3.41) | 0.57 (0.12, 2.59) | 1.40 (0.22, 8.89) | 8.76 | 4.60 (0.62, 33.85) |
| **Gender** | | | | | |
| Female | Ref | Ref | Ref | Ref | Ref |
| Male | 0.80 (0.34, 1.87) | 0.46 (0.15, 1.40) | 0.61 (0.19, 1.98) | 0.62 (0.16, 2.42) | 1.00 (0.15.,6.71) |
| **Education** | | | | | |
| Secondary | Ref | Ref | Ref | Ref | Ref |
| Bachelor’s Degree | 1.40 (0.48, 4.05) | 7012474 | 3.17 (0.60, 16.74) | 3.55 (0.39, 32.66) | 2.32 (0.20, 26.40) |
| Graduate Degree | 1.09 (0.30, 3.93) | 1.99 | 0.38 (0.26, 5.45) | 4.18 (0.37, 47.23) | 2.34 |
