# Supplementary material for: What message appeal and messenger are most persuasive for COVID-19 vaccine uptake: Results from a 5-country survey in India, Indonesia, Kenya, Nigeria, and Ukraine
Source: PLoS One. 2022 Sep 21;17(9):e0274966. doi: 10.1371/journal.pone.0274966 (PMC9491563; doi:10.1371/journal.pone.0274966)
Supplement: S5 Table — (DOCX) [file pone.0274966.s005.docx]

**S5 Table: Ukraine relative risk ratios of ad preference by vaccine hesitancy status and participant characteristics using multivariable multinomial logistic regression modeling** (n=155)*

|  | ***Adjusted relative risk ratios (95% CI)*** | | | | | |
| --- | --- | --- | --- | --- | --- | --- |
|  | **Health Outcome**  **Peer** | **Economic**  **Healthcare provider** | **Economic**  **Peer** | **Social norm**  **Healthcare provider** | | **Social norm**  **Peer** |
| **Vaccine hesitancy** | | | | | | |
| Lower | Ref | Ref | Ref | Ref | Ref | |
| Higher | 1.53 (0.35, 6.63) | 1.84 (0.44, 7.66) | 4865011 | 0.63 (0.22, 1.87) | 1.06 (0.23, 4.83) | |
| **Age** | | | | | | |
| <40 | Ref | Ref | Ref | Ref | Ref | |
| 40+ | 0.57 (0.16, 2.06) | 0.79 (0.26, 2.44) | 2.87 (0.70, 11.86) | 1.77 (0.68, 4.62) | 1.53 (0.41, 5.63) | |
| **Gender** | | | | | | |
| Female | Ref | Ref | Ref | Ref | Ref | |
| Male | 0.55 (0.19, 1.58) | 0.77 (0.29, 2.00) | 0.50 (0.12, 2.08) | 0.95 (0.38, 2.35) | **0.21 (0.05, 0.87)** | |
| **Education** | | | | | | |
| Secondary | Ref | Ref | Ref | Ref | Ref | |
| Bachelor’s Degree | 9490045 | 3.50 (0.81, 15.04) | 1.13 (0.16, 8.12) | 1.30 (0.37, 4.57) | 0.49 (0.07, 3.47) | |
| Graduate Degree | 5220313 | 2.51 (0.58, 10.85) | 1.58 (0.25, 9.83) | 1.43 (0.47, 5.07) | 1.84 (0.40, 8.43) | |
| * Reference category: health outcome / healthcare provider ad | | | | | | |
